# Biosimilar insulin concepts

**DOI:** 10.1111/1753-0407.13267

**Published:** 2022-03-28

**Authors:** Zachary T. Bloomgarden

**Affiliations:** ^1^ Icahn School of Medicine Mount Sinai New York NY USA

**FIGURE 1:**
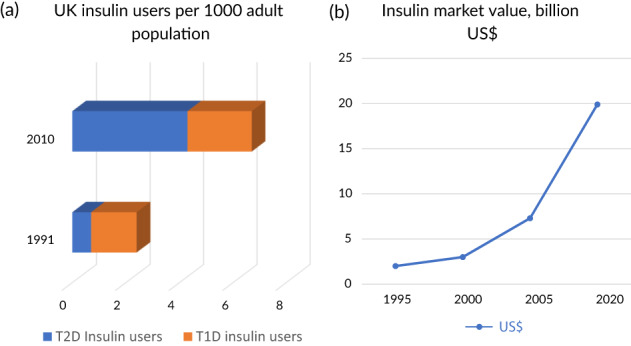
(A) UK insulin users per 1000 adult population, 1991 and 2010. (B) Total annual insulin market value, $US billions, 1995–2020. Redrawn from data in^1^ T1D, type 1 diabetes; T2D, type 2 diabetes

**FIGURE 2:**
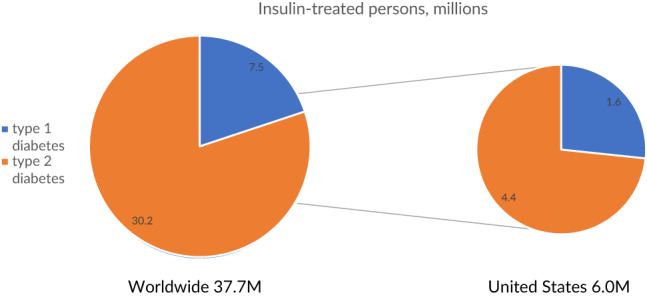
Estimated current number of insulin users with T1D and with T2D, worldwide and United States. Drawn from data in^2^, ^3^, ^4^, ^5^, ^6^ T1D, type 1 diabetes; T2D, type 2 diabetes

Insulin use has dramatically increased over the past 30 years, in part because of the increasing realization of its role in the treatment of type 2 diabetes (T2D) (Figure 1A).^1^ Worldwide, the prevalence of type 1 diabetes (T1D) is 0.95 per thousand persons,^2^ for a total of approximately 7.5 million persons who require insulin in multiple daily dosages out of the world population of 7.9 billion.^3^ In addition, an estimated 30.2 million persons with T2D are treated with insulin globally, using more than 500 billion units of insulin per year,^4^ at a cost of some US$20 billion (Figure 1B). In the United States, 1.6 million persons have T1D^5^ of the total of 6.0 million people with diabetes who use insulin^6^ (Figure 2).

Many people with diabetes use biosynthetic human insulin (BHI) produced by recombinant DNA technology. In 1996 the first rapid‐acting insulin analog became available. This molecule, insulin lispro, is formulated by interchanging the positions of the amino acids lysine and proline at the end of the B chain of the insulin molecule, resulting in weaker self‐association than BHI, with consequent greater rapidity of onset and shorter duration of action.^7^ Four years later, in 2020, the first long‐acting analog was approved, insulin glargine, with addition of two arginine residues to the B‐chain and substitution of one of the asparagine residues of the A‐chain by glycine, allowing the molecule to be formulated as an acidic solution that is slowly and evenly absorbed.^8^ Although some consider human insulin preparations to be similar in efficacy and benefit to insulin analogs,^9^ there is reasonable evidence suggesting that insulin analogs are preferable. Compared with (eutral protamine Hagedorn (NPH), long‐acting insulin analog treatment is associated with significant reduction in hypoglycemia,^10^ as well as with evidence of improvement in glycemic control.^11^ Postprandial hyperglycemia, nocturnal hypoglycemia, and HbA1c are reduced to a greater degree in people with T1D receiving insulin aspart^12^ and other rapid‐acting analogs^13^ rather than BHI, and reduction in postprandial hyperglycemia is seen in people with T2D comparing these two prandial insulin preparations.^12^ There has therefore been great interest in approaches to increase availability of analog insulin preparations for people with diabetes. Part of the impetus to the development of such products has been the high cost of insulin analogs, with the average price of rapid‐acting and long‐acting insulin analogs particularly high in the United States, between 8‐ and 20‐fold that in other developed countries,^14^ costing nearly US$6000 per insulin‐treated person annually, with estimates that widespread availability of biosimilar insulin analogs could reduce this cost more than 50‐fold.^15^ As a recent commentator observed, “The WHO added long‐acting analogues to its Essential Medicines List in its 2021 revision… Some people undoubtedly benefit from their use… Cost apart, there is no very good reason not to use them.”^1^

The US Food and Drug Administration (FDA) has suggested three levels of similarity that can be used in characterizing complex biologic pharmaceuticals such as insulin, with the goal of helping prescribers to offer appropriate biosimilar insulin analogs to people with diabetes treated with existing more expensive products. The FDA defines a follow‐on biologic as one sufficiently similar to the original FDA‐approved biologic to permit reliance on existing scientific knowledge about safety/effectiveness, noting that this is determined on a case‐by‐case basis. A biosimilar is a follow‐on biologic that is “highly similar to the reference product notwithstanding minor differences in clinically inactive components,” and having “no clinically meaningful differences from reference product in safety, purity, and potency.” Finally, the FDA has proposed that a biosimilar be termed “interchangeable” if the prescriber can expect the same clinical result as with the reference product in any given patient, and if “the risk in terms of safety or diminished efficacy of alternating or switching between use of the [biosimilar] and the reference product is not greater than the risk of using the reference product without such alternation or switch.”^16^

The distinctions made between the three levels of similarity may be necessary for regulatory purposes but are clearly somewhat arbitrary. What is the difference between “sufficiently similar” and “highly similar”? In determining “the risk of using the reference product,.” one should consider whether the biologic action of existing insulin preparations is itself consistent. For example, the product monograph for the Lantus formulation of insulin glargine notes that the time between injection and the end of pharmacological effect ranges from 10.8 to >24.0 h for insulin glargine. Furthermore, for any given individual with diabetes there is great variability in insulin requirements from one day to the next. A study of 32 persons with T1D who were treated with a closed‐loop insulin infusion for 12 weeks showed that the coefficient of variation (CV) of the overnight insulin requirement relative to baseline was 31%.^17^ Recall that the CV is the SD divided by the mean, and that approximately 68% of a normally distributed data set is within 1 SD from the mean, so that the findings of this study suggest that 15.5% of the time the insulin requirement will be 34% more than the mean insulin requirement, and 15.5% of the time the insulin requirement will be 34% less. For 67 hospitalized persons with T2D who were treated with a closed‐loop insulin infusion for 4–15 days, the overnight CV of the insulin infusion requirement was more than 50%.^18^ In a study of 2342 persons with T2D initiating a variety of insulin regimens, the CV of intraday self‐monitored blood glucose (SMBG) was the factor having the greatest influence on hypoglycemia,^19^ suggesting, as one would expect, that this measure of variability has a strong relationship to this important risk associated with insulin treatment.

There is evidence that the glucose CV remains high in persons with diabetes treated with insulin glargine. An observational study of 1167 hospitalized persons with T1D and T2D treated with insulin glargine showed a glucose CV of 34.8%,^20^an outpatient continuous glucose monitoring study of 17 persons with T2D treated with insulin glargine 0.2 U/kg/day with 24‐h mean glucose 9.5 mmol/L showed glucose CV 26.8%,^21^ and a study based on SMBG measured by 88 persons with T1D using insulin glargine as basal insulin showed the CV of the fasting and predinner glucose was 41.1% and 40.1%.^22^ In considering the applicability to insulin prescription of the three levels of similarity defined by the FDA for biologics, then, one should realize that a given dose of insulin has highly variable expected action, reminding us of the dictum of Elliot Joslin in 1923, just 2 years after the discovery of insulin: “Insulin is a remedy primarily for the wise and not for the foolish, whether they be patients or doctors.”^23^

Given these considerations, studies of the effectiveness of a variety of biosimilar insulin preparations do show satisfactory results. A meta‐analysis of 14 randomized controlled trials with 6188 participants receiving long‐ and short‐acting biosimilar insulin formulations in comparison to their reference products showed no significant difference in HbA1c or fasting glucose change either at 26 or at 52 weeks, or in total hypoglycemia or severe hypoglycemia, without difference in these outcomes for study participants with T1D or T2D analyzed separately.^24^ Looking at specific biosimilar insulin products, SAR342434 insulin lispro was studied in 500 persons with T1D^25^ and 497 with T2D,^26^ showing no differences in lispro or glargine doses, A1c, fasting plasma glucose, SMBG, hypoglycemia, or weight change, although a “switch study” was not performed.^27^ A study of 597 persons with T1D or insulin‐treated T2D randomized to the reference insulin aspart or biosimilar SAR341402 insulin aspart showed no difference in hypoglycemia, HbA1c, or total prandial insulin requirement.^28^ Biosimilar insulin glargine LY2963016 showed identical HbA1c and SMBG day‐profile glucose levels to those with the reference insulin glargine both with T1D^29^ and T2D.^30^ Similar findings were reported in two studies of biosimilar Basalin insulin glargine in a continuous glucose monitoring study of 100 persons with T2D^31^ and in a study of HbA1c and fasting and postprandial glucose changes in 133 persons with T2D.^32^ Among 113 C‐peptide negative persons with T1D undergoing a Biostator clamp study with biosimilar MYL‐1501D Insulin glargine, 30‐h plasma levels of the main M1 product of glargine metabolism as well as the 30‐h glucose infusion rate curve were identical to those with reference insulin glargine. To specifically address the FDA criterion of interchangeability, a switch study was done with MYL‐1501D insulin glargine administration for 12 weeks, then reference glargine for 12 weeks, and then again administering MYL‐1501D for 12 weeks, or, for a comparator, administering reference glargine for 36 weeks, showing stable HbA1c and basal insulin doses.^33^ Based on this study and the subsequent FDA designation that this preparation can be considered interchangeable with the reference insulin glargine, a new nomenclature has been designated for the product as insulin glargine‐yfgn.^34^

An issue to which the FDA has paid great attention is that insulin is immunogenicity, and in the meta‐analysis^24^ as well as the specific studies described above no difference was reported in insulin antibody formation between reference products and any of the biosimilar insulin preparations. The one case report of a hypersensitivity reaction to a biosimilar insulin glargine manufactured in Mexico by Landsteiner Scientific^35^ may reflect a lack of product preparation control in a specific batch, pointing to the importance of great care in pharmaceutical practice in the production of the complex biosimilar insulin molecule. Yet another issue, however, and one that may be much more important to insulin‐treated people with diabetes than differences in pharmacokinetics or pharmacodynamics, is the role of devices. Insulin analogs are often dispensed in disposable prefilled pen‐injectors, with unique color coding, tactile identifiers, and specific dose ranges and device capacities, requiring specific injection force, and many other characteristics to which a person using the product may become accustomed. As people with diabetes become familiar with a particular pen device, changing the pen might be more of a concern for them than the change in the insulin analog product. The designation that a product is “interchangeable” may then lead to erroneous dosing with the potential to be more serious than that of differences in precise dose equivalence.^36^

There is, then, a strong rationale for encouraging biosimilar insulin development, and the effectiveness of biosimilar insulin analog preparations developed to date appears satisfactory. The concept of stressing interchangeability as a criterion may, however, not be as important clinically as has been thought by regulatory authorities, given the marked variation in day‐to‐day insulin action in persons treated with the products currently in use, and issues with different devices of supposedly interchangeable insulin preparations are quite likely to cause clinical issues. Careful attention to this and other potential safety issues of biosimilar insulin preparations will be of the utmost importance in ensuring that these important products are available to the large number of persons who need affordable, effective insulin treatment.
